# Interfaces between Model Co-W-C Alloys with Various Carbon Contents and Tungsten Carbide

**DOI:** 10.3390/ma11030404

**Published:** 2018-03-09

**Authors:** Igor Konyashin, Alexander Zaitsev, Alexander Meledin, Joachim Mayer, Pavel Loginov, Evgeny Levashov, Bernd Ries

**Affiliations:** 1 Department of Powder Metallurgy and Functional Coatings, National University of Science and Technology “MISiS”, Leninsky pr. 4, 119049 Moscow, Russia; aazaitsev@bk.ru (A.Z.); pavel.loginov.misis@list.ru (P.L.); levashov@shs.misis.ru (E.L.); 2 Element Six GmbH, Städeweg 12-24, 36151 Burghaun, Germany; bernd.ries@e6.com; 3 Central Facility for Electron Microscopy (GFE), RWTH Aachen University, Ahornstrasse 55, D-52074 Aachen, Germany; meledin@gfe.rwth-aachen.de (A.M.); mayer@gfe.rwth-aachen.de (J.M.); 4 Ernst Ruska-Centre for Microscopy and Spectroscopy with Electrons Research Centre Juelich, D-52425 Juelich, Germany

**Keywords:** cemented carbides, nanoparticles, model binder alloys, tungsten carbide, carbon content

## Abstract

Interfaces between alloys simulating binders in WC-Co cemented carbides and tungsten carbide were examined on the micro-, nano-, and atomic-scale. The precipitation of fine WC grains and η-phase occurs at the interface of the alloy with the low carbon content. The precipitation of such grains almost does not occur in the alloy with the medium-low carbon content and does not take place in the alloy with the high carbon content. The formation of Co nanoparticles in the binder alloy with the medium-low carbon content was established. Interfaces in the alloy with the medium-low carbon content characterized by complete wetting with respect to WC and with the high carbon content characterized by incomplete wetting were examined at an atomic scale. The absence of any additional phases or carbon segregations at both of the interfaces was established. Thus, the phenomenon of incomplete wetting of WC by liquid binders with high carbon contents is presumably related to special features of the Co-based binder alloys oversaturated with carbon at sintering temperatures.

## 1. Introduction

Interfaces and grain boundaries in metal-ceramic composites, particularly WC-Co cemented carbides, play an important role with respect to obtaining high mechanical and performance properties of such materials. The state of WC-Co grain boundaries in cemented carbides is thought to determine a unique combination of different properties of such materials. Nevertheless, there is almost no information in the literature on WC-Co interfaces in WC-Co materials that are not containing grain growth inhibitors at an atomic level by use of high-resolution transmission electron microscopy (HRTEM) studies. Such studies are complicated due to difficulties with respect to finding in transmission electron microscopy (TEM) WC-Co interfaces with both WC and Co grains simultaneously in a zone axis orientation.

Also, the interaction between Co-based binders and WC grains in WC-Co cemented carbides on the initial stages of liquid-phase sintering plays an important role with respect to obtaining cemented carbides with proper microstructures and properties. In the literature, there is no information on interfaces between tungsten carbide grains and Co-based binders having various carbon contents when liquid-phase sintering is interrupted in several seconds after the liquid phase formation.

The interaction between Co-based binders and WC grains on early stages of liquid-phase sintering can be strongly affected by the carbon content in the binders, which was established in refs. [1,2]. In ref. [1], it was found that the wettability of tungsten carbide by liquid Co-based binders is complete at low carbon contents and incomplete at high carbon contents. However, a reason for the incomplete wettability at high carbon contents is not understood so far. It can be supposed that the reason is related either to the segregation of carbon atoms at the WC/binder interface or to special features the Co-based binders oversaturated with carbon at sintering temperatures. In ref. [3], a dependence of the wetting efficiency on the carbon chemical potential in the binder phase is predicted, which can explain the better wetting of WC that was observed experimentally at low carbon contents.

The interaction between Co-based binders with different carbon contents and WC grains on early stages of liquid-phase sintering is thought to affect the well-known phenomenon of segregation of different grain growth inhibitors (vanadium carbide, chromium carbide, etc.) at WC/Co interfaces in cemented carbides [4,5,6]. Also, the interaction between Co-based binders and WC can lead to the formation of complexions in straight WC-Co cemented carbides that are not containing grain growth inhibitors [7].

There is no information in the literature on the atomic structure of WC-Co interfaces obtained by use of Co-based binder alloys with various carbon content. Meanwhile, understanding the nature of WC-Co interfaces can clarify the well-known phenomenon of the liquid binder migration from the WC-Co regions with high carbon contents into the regions with low carbon contents. This phenomenon is widely employed for the fabrication of functionally graded cemented carbides having a surface layer with a significantly reduced Co content and a core region with a high Co content [8,9,10,11]. It was suggested in refs. [1,2] that this phenomenon occurs as a result of different wettability rates of WC by binders with various carbon contents. However, a mechanism explaining the different wettability rates with respect to the WC/Co interfaces on the micro-, nano-, and atomic-level is not understood.

The major objective of this work was to examine interfaces between model Co-W-C binder alloys with various carbon contents and polycrystalline tungsten carbide samples after short sintering at temperatures of slightly above the corresponding melting points.

## 2. Materials and Methods

Powders of the model alloys containing 65 wt % Co, corresponding to the eutectic composition in the pseudo-binary phase diagram W-Co-C, with various amounts of tungsten metal and carbon black added were mixed in a Turbular mixer. A WC powder with a mean grain size of 0.8 µm and carbon content of 6.13 wt % and a Co powder with mean grain size of nearly 1 µm were employed. Three binder alloys with a low carbon content (1.53 wt % C), medium carbon content (2.61 wt % C) and high carbon content (4.02 wt % C) were made. All the alloys were melted in alumina crucibles in a vacuum at a temperature of 1500 °C for 1 h. As a result, the Co-based model binder alloys contain some dissolved carbon and tungsten, thus simulating the real binders in WC-Co that was cemented carbides. Small samples having a cylindrical shape were cut off from the melted samples and employed for experiments on examining the interaction between the model alloys and polycrystalline WC samples.

The polycrystalline WC samples were made from a near-nano WC powder with the WC mean grain size of 0.2 µm. They were first subjected to hot pressing at temperature of 2000 °C and pressure of 60 MPa for 20 min, followed by hot isostatic pressing in Ar at a temperature of 2100 °C and pressure of 100 bar for 1 h. The samples have a residual porosity of roughly 2%.

Experiments on examining the interaction between the model binder alloys and the polycrystalline WC samples were performed in a vacuum furnace. The cylindrical samples of the alloys were located on the surface of the polycrystalline WC samples and slowly heated up to temperatures slightly above their melting points and were subjected to liquid-phase sintering for 35 s. The melting points were established by the DTA studies by use of the STA 449 F1 and STA 429 instruments (Netzsch, Selb, Germany).

Cross-sections of the WC/binder samples after liquid-phase sintering were examined on a light microscope and a scanning electron microscope (SEM, S-3400N, Hitachi, Tokyo, Japan), equipped with a device for energy-dispersive X-ray microanalysis (EDX). X-ray diffraction (XRD) was performed by the use of a DRON-3 X-ray diffractometer (Techoborudovanie, Moscow, Russia) using the Cu-Kα radiation. For transmission electron microscopy (TEM) studies, cross-sectional lamellae of the model alloy/WC interfaces were prepared by the focused ion beam (FIB) technique on a FEI Quanta 200 3D FIB instrument (FEI, Hillsboro, OR, USA). High resolution TEM (HRTEM), high angle annular dark field scanning TEM (HAADF-STEM) imaging, and selected area electron diffraction (SAED) were performed on a JEOL-JEM-2100 transmission (JEOL, Tokyo, Japan) electron microscope as well as on the both probe and image aberration corrected FEI Titan 80–300 transmission electron microscopes operated at 200 kV and 300 kV.

## 3. Results and Discussion

**Figure 1:**
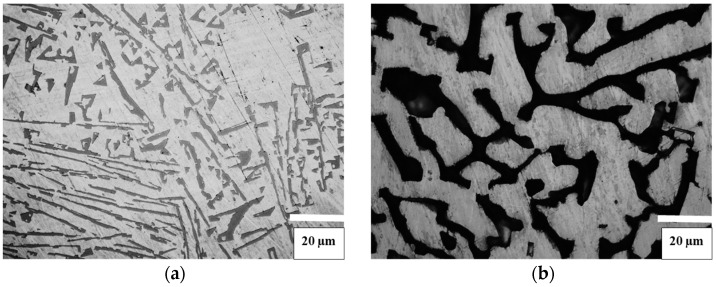

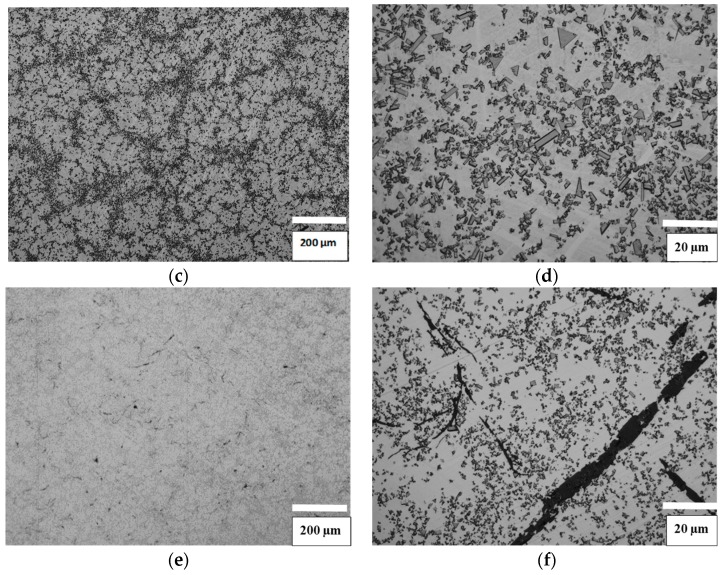
Microstructures of model binder alloys with various carbon contents after their original sintering at a temperature of 1500 °C: (**a**,**b**) With a low carbon content (**a**) needle-like WC grains and (**b**) Dendritic η-phase crystals); (**c**,**d**) With a medium-low carbon content at different magnifications; (**e**,**f**) With a high carbon content at different magnifications. All of the micrographs except for (**e**) are obtained after etching in the Murakami reagent.

Figure 1 shows microstructures of the three alloys with various carbon contents after their initial sintering at a temperature of 1500 °C. According to XRD results and microstructural examination results, the microstructure of the alloy with the low carbon content (Figure 1a,b) comprises η-phase, WC in the form of needle-like grains, and a Co-based binder. It was established that melting of this alloy starts at 1370 °C and ends at 1399 °C. The microstructure of the alloy with the medium-low carbon content (Figure 1c,d) comprises only WC grains surrounded by a Co-based binder matrix. It was established that melting of this alloy starts at 1332 °C and ends at 1366 °C. According to the XRD results the Co-based binder phase consists of fcc Co. The microstructure of the alloy with the high carbon content (Figure 1e,f) comprises WC, Co, and graphite in form of needle-like precipitates. It was established that melting of this alloy starts at 1299 °C and ends at 1330 °C. The Co-based binder phase of this alloy comprises a mixture of fcc Co and hcp Co.

The precipitation behavior and the phase constitution of the three model binder alloys can be related to the results of thermodynamic calculations of the W-Co-C phase diagram at 65 wt % Co, which were obtained in ref. [1]. Particularly, special features of the microstructure of the model alloy with the low carbon content can be understood when taking into account that it crystallizes from the region of the phase diagram, where only the liquid phase and no solid WC phase are present at the sintering temperature (1500 °C). The microstructures of the alloys with medium-low and high carbon contents are obtained as a result of cooling down from the region of the phase diagram, where both the Co-based liquid phase and the solid WC phase coexist at the sintering temperature of 1500 °C.

**Figure 2:**
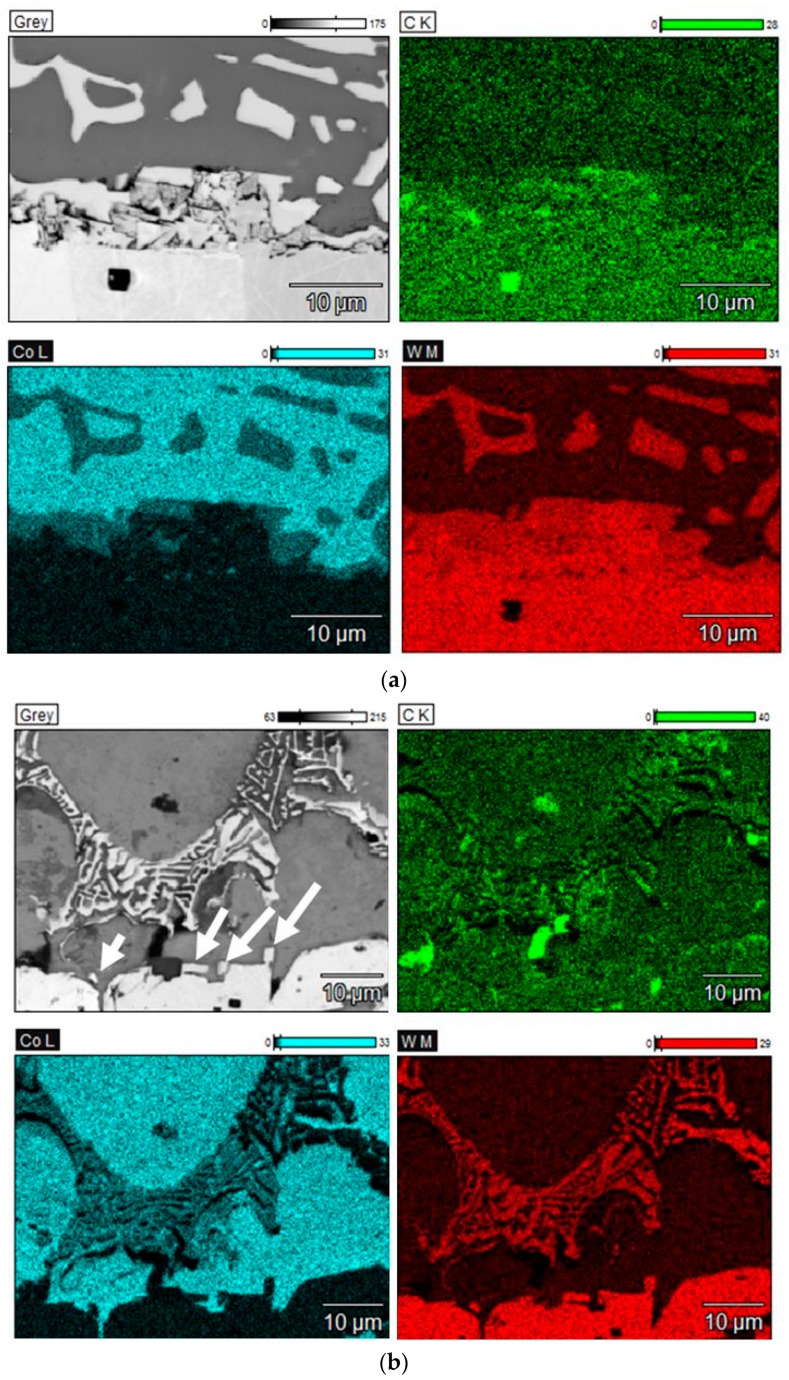

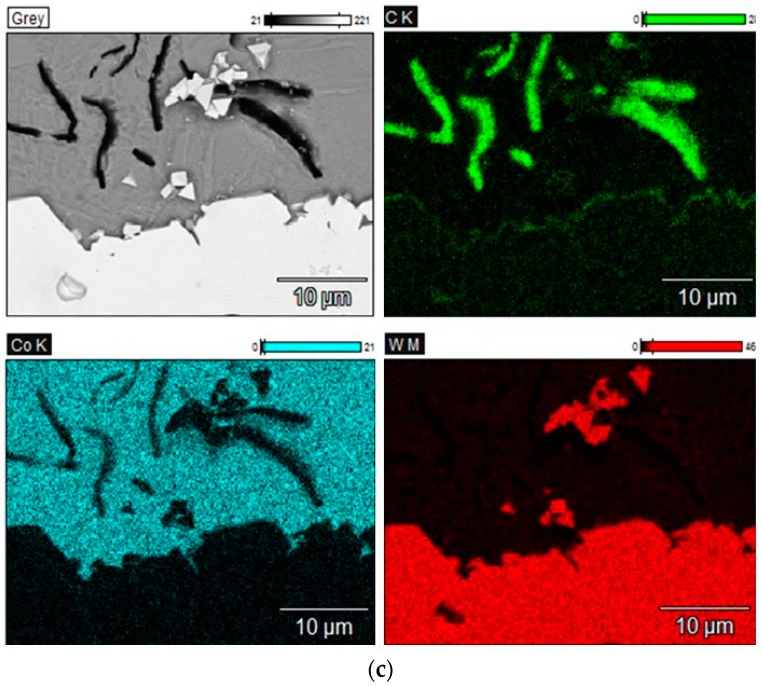
Scanning electron microscope (SEM) images and elemental maps of C, Co, and W in the interface region between the model binder alloys and polycrystalline WC samples: (**a**) Alloy with the low carbon content; (**b**) Alloy with the medium-low carbon content (precipitates of fine WC grains are indicated by arrows); (**c**) Alloy with the high carbon content.

Figure 2 shows SEM images and EDX elemental maps of the WC/Co interfaces.

As one can see in Figure 2a small grains precipitate from the alloy with the low carbon content on the surface of the polycrystalline tungsten carbide sample. These precipitates consist of WC and η-type phase, as some of them do not contain any Co and some of them contain some Co. The precipitation of the fine grains in the initial stage of liquid-phase sintering, which was not described in the literature before, is presumably related to the well-known fact that the model alloy with the low carbon content is oversaturated with tungsten metal [12]. As a result of the interaction of such a binder alloy that was oversaturated with tungsten with the polycrystalline tungsten carbide sample, the excess of tungsten presumably reacts with the carbon containing in the surface layer of the polycrystalline sample leading to its partial decarburization and formation of the fine grains of WC and η-type phase.

Figure 2b shows a SEM image of the interface region after melting the alloy with the medium-low carbon content on the surface of the polycrystalline tungsten carbide sample. As it can be seen in Figure 2b, the interface region comprises very few precipitates of small WC grains deposited directly on the surface of the polycrystalline tungsten carbide sample. This is presumably related to the fact that this model alloy comprises significantly less dissolved tungsten metal than the alloy with the low carbon content.

Figure 2c shows a SEM image of the interface region after melting the alloy with the high carbon content on the surface of the polycrystalline tungsten carbide sample. One can see that there are no precipitates that are similar to those forming in the case of the model alloy with the low carbon content, and there are also no inclusions of free carbon at the Co/WC interface.

**Figure 3:**
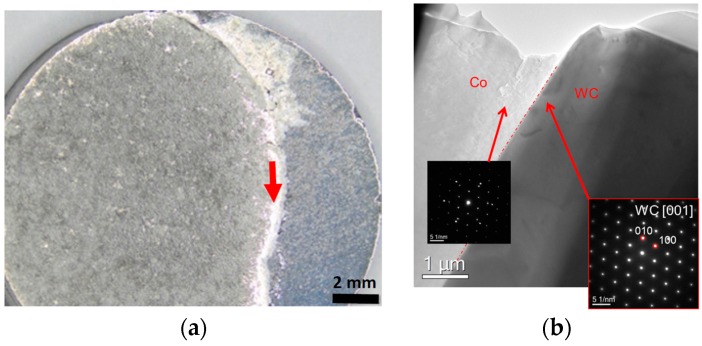
WC-Co interfaces in the sample obtained after melting the alloy with the medium-low carbon content on the WC surface: (**a**) The sample appearance with an arrow indicating where a focused ion beam (FIB) lamella was cut off from the interface region (note that the sample is characterized by complete wetting); (**b**) Interface nano-structure with insets indicating selected area electron diffraction (SAED) patterns.

Figure 3a shows the appearance of the polycrystalline WC sample with a film of the binder alloy with the medium-low carbon content after its melting and spreading over the WC sample. The wetting angle is close to 0 in this case indicating complete wetting. A FIB lamella was cut off from the region of the sample indicated by an arrow in Figure 3a. Figure 3b shows the nano-structure of the WC/Co interface for the model binder alloy with the medium-low carbon content with insets indicating SAED patterns. It was not possible to index the binder phase as fcc or hcp Co by the use of electron diffraction, however, XRD results indicate that Co is present in this sample as the cubic modification.

**Figure 4:**
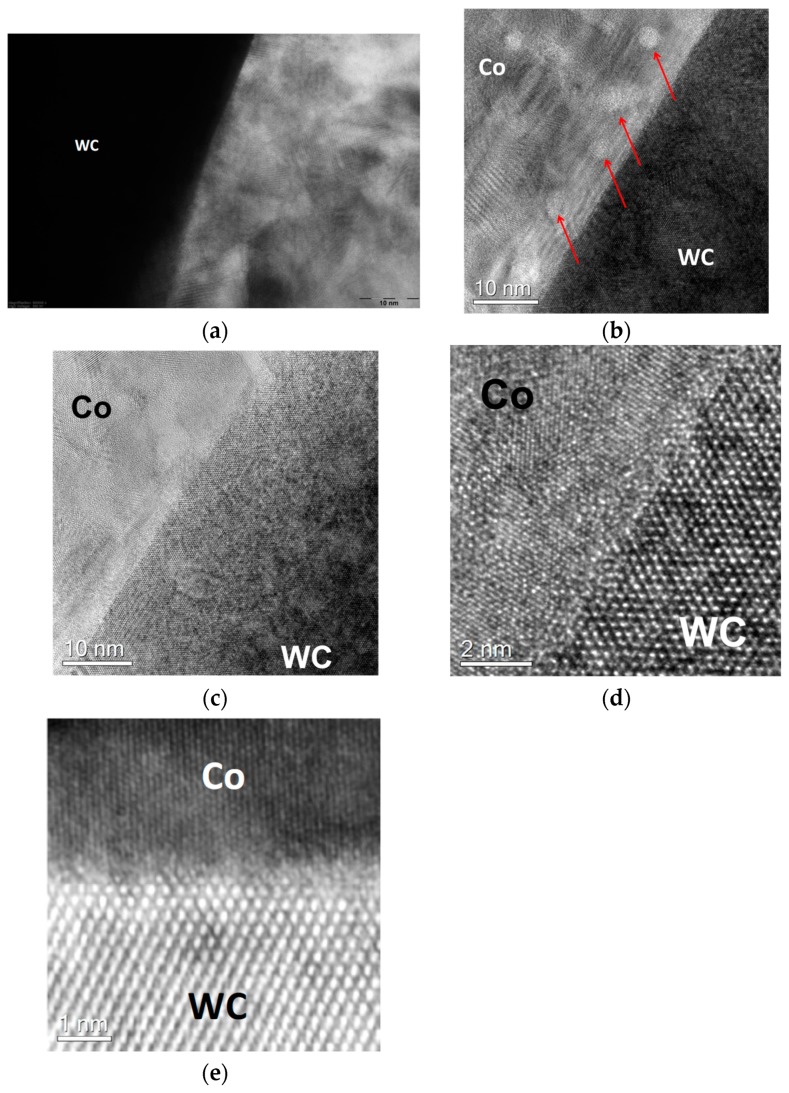
WC-Co interfaces in the sample obtained after melting the alloy with the medium-low carbon content on the WC surface: (**a**) Binder nano-structure near the interface (JEOL JEM-2100); (**b**–**d**) Aberration corrected high-resolution transmission electron (HRTEM) images of the interface at different magnifications (nanoparticles in the Co-based alloy are indicated by arrows), (**e**) High Angle Annular Dark Field Scanning TEM (HAADF STEM) after gamma-correction and high-pass filtering (FEI Titan 80–300).

Figure 4a–e show the WC/Co interface for the binder alloy with the medium-low carbon content at an atomic scale. In Figure 4a,b, it can be seen that the Co-based alloy comprises Co nanoparticles of several nanometer in size having different crystal lattice orientations, which was established for the first time in the present work. It should be mentioned that the mean grain size of the Co-based binders in industrial cemented carbides after conventional sintering is of the order of 100 µm or larger [12]. The presence of such Co nanoparticles after the short liquid-phase sintering indicates that the Co-based binder can be nanostructured. This can be a result of the presence of the liquid phase for a short time, followed by its fast cooling. The nanoparticles presumably form due to the formation of a great number of solid Co nuclei in the whole volume of the liquid model alloy, which are maintained due to the rapid binder solidification. According to ref. [12], binder alloys of cemented carbides with low carbon contents comprise relatively much dissolved tungsten (up to 20 wt %), which can play an important role with respect to formation of such Co nanoparticles.

As it can be seen Figure 4c–e, the crystal lattices of Co and WC are obviously incoherent and the interface is clean and free of any inclusions, defects, or disorders, which explains the complete wetting of WC by the Co-based binders with the medium-low carbon contents [1,2].

**Figure 5:**
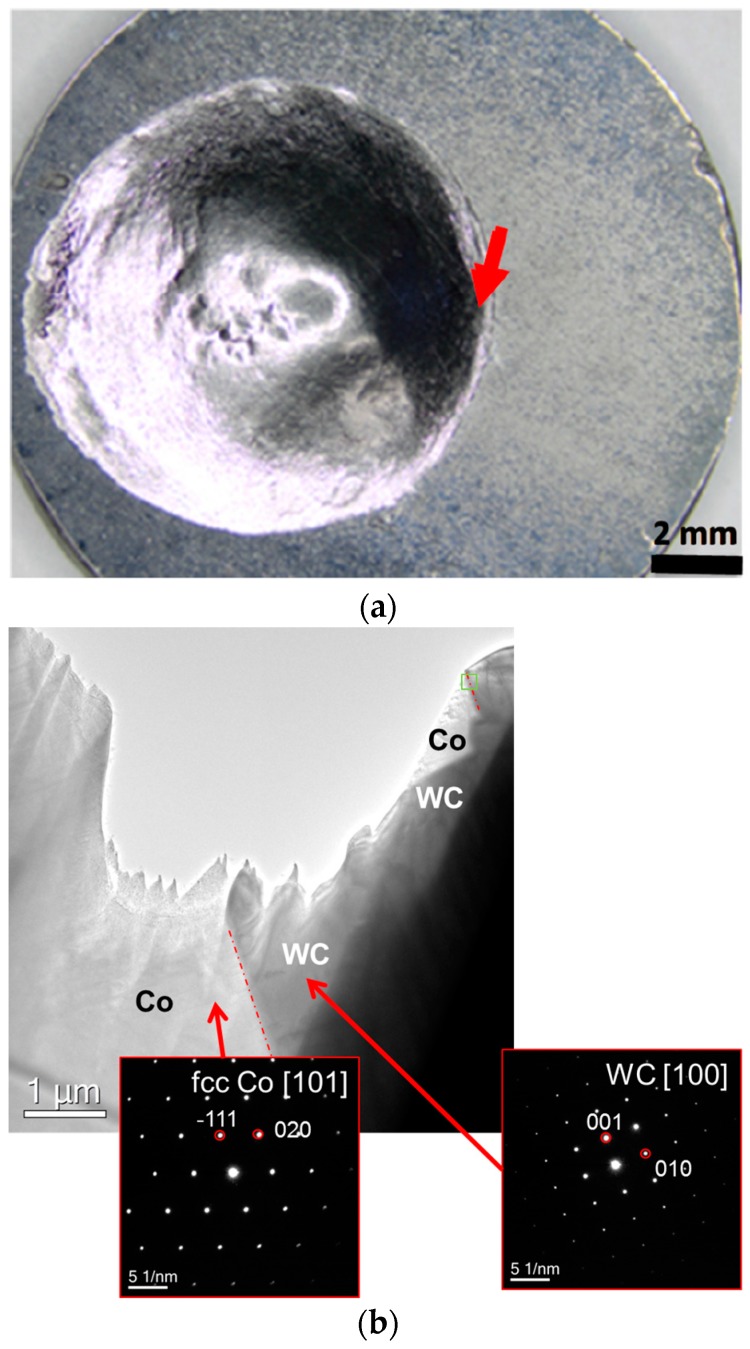
WC-Co interfaces in the sample that was obtained after melting the alloy with the high carbon content on the WC surface: (**a**) The sample appearance with an arrow indicating where a FIB lamella was cut off from the interface region (note that the sample is characterized by incomplete wetting); (**b**) Interface nano-structure with insets indicating SAED patterns.

Figure 5a shows the polycrystalline WC sample with a droplet of the binder model alloy with the high carbon content after its melting followed by solidification. The wetting angle is equal to roughly 15° in this case indicating that wetting is incomplete. A FIB lamella was cut off from the region of the sample indicated by an arrow in Figure 5a. Figure 5b shows the interface nano-structure for the binder model alloy with the high carbon content with insets indicating SAED patterns, which provide evidence that Co is present in the fcc crystal modification.

**Figure 6:**
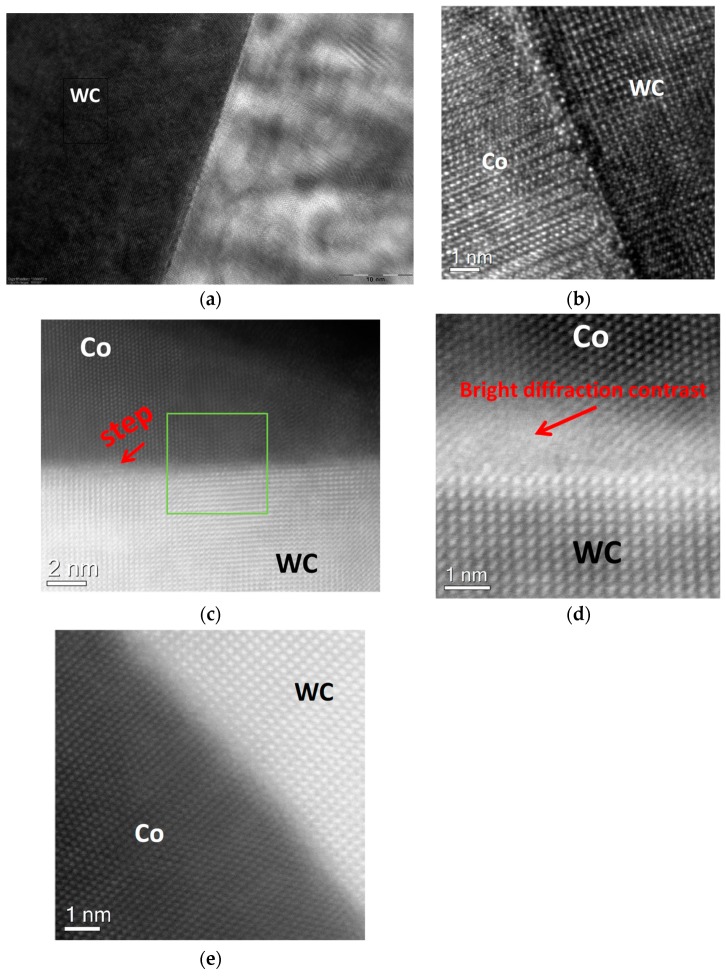
WC-Co interfaces in the sample obtained after melting the alloy with the high carbon content on the WC surface: (**a**) Binder nano-structure near the interface (JEOL JEM-2100); (**b**) HRTEM image of the interface (JEOL JEM-2100); (**c**) HAADF STEM (Z-contrast) image of the interface; (**d**) ADF STEM (both diffraction- and Z-contrasts) image of the interface; (**e**) Fourier-filtered high-resolution STEM image of the interface (FEI Titan 80–300).

Figure 6 shows HRTEM image of the WC/Co interface between the binder model alloy with the high carbon content and tungsten carbide. The interface between WC and the Co-based binder with the high carbon content shown in Figure 6a,b is characterized by defects, disorders, and the presence of a dark contrast layer at the interface. The Co grain shown in Figure 6a appears to be highly twinned. In contrast to the sample with the medium-low carbon content, there are no nanoparticles in the Co-based alloy with the high carbon content, which is presumably a result of oversaturating this alloy with carbon, resulting in low concentrations of tungsten being dissolved in this model alloy.

Additional high-resolution STEM studies of the WC-Co interface were carried out in order to clarify the atomic structure of the interface, which cannot be clearly established by use of the high-resolution TEM (Figure 6a,b). Figure 6c shows a HAADF STEM (Z-contrast) image of the interface and Figure 6d shows an ADF STEM (both diffraction- and Z-contrasts) image of the interface. The WC surface roughness made the investigation of the WC-Co interface difficult. However, it was possible to find a region with a relatively sharp WC-Co interface. As one can see in the HAADF STEM (Z-contrast) image (Figure 6c), there is no dark contrast layer at the WC/Co interface providing evidence for the absence of carbon inclusions, precipitations, or segregated carbon atoms at the interface. The Co-layer adjacent to the WC-Co interface is formed by a twinned single crystal. The bright diffraction contrast indicates the strained dislocation regions in the Co layer near the WC/Co interface shown in Figure 6d,e. Therefore, incomplete wetting of WC by the model binder alloy with the high carbon content is presumably related to the special features of the Co-based alloys oversaturated with carbon, which corresponds well to the theoretical prediction that is obtained in ref. [3].

## 4. Conclusions

A phenomenon of precipitating fine grains of WC and η-phase was for the first time observed in the interface region of the binder model alloy with the low carbon content. This phenomenon almost does not occur in the model alloy with the medium-low carbon content and does not occur in the model alloy with the high carbon content. The formation of Co nanoparticles in the binder model alloy with the medium-low carbon content near the WC/Co interface was established for the first time. The WC/Co interface is free of any inclusions, defects, or disorders at an atomic scale in the case of the binder alloy with the medium-low carbon content. The interface between WC and the binder model alloy with the high carbon content is free of any carbon segregation or precipitation at an atomic scale. Therefore, it is thought that the incomplete wetting of WC by liquid Co-based binders with high carbon contents in cemented carbides is related to special features of the binder alloys that were oversaturated with carbon at sintering temperatures.
